# *wigExplorer*, a BioJS component to visualise wig data

**DOI:** 10.12688/f1000research.3-53.v3

**Published:** 2016-08-09

**Authors:** Anil S. Thanki, Rafael C. Jimenez, Gemy G. Kaithakottil, Manuel Corpas, Robert P. Davey

**Affiliations:** 1 Earlham Institute, Norwich Research Park, Norwich, NR4 7UH, UK; 2 European Bioinformatics Institute, Hinxton, Cambridge, CB10 1SD, UK

**Keywords:** BioJS, data visualisation, genome browsers

## Abstract

**Summary:** *wigExplorer* is a BioJS component whose main purpose is to provide a platform for visualisation of wig-formatted data. Wig files are extensively used by genome browsers such as the UCSC Genome Browser. *wigExplorer *follows the BioJS standard specification, requiring a simple configuration and installation. *wigExplorer* provides an easy way to navigate the visible region of the canvas and allows interaction with other components via predefined events.

**Availability:** http://biojs.io/d/biojs-vis-wigexplorer ; http://dx.doi.org/10.5281/zenodo.8516

## Introduction

Numerous web applications exist for visualisation of biological data. Data can be prepared for visualisation using a variety of formats, one of which is the widely used wiggle (wig) file. A wiggle file contains text that defines either a feature or a data track. The wiggle format was developed by the UCSC genome browser ^1^ and then quickly adopted by other initiatives ^2, 3^. Web applications such as genome browsers rely heavily on JavaScript, a popular language for processing and rendering client-side information in a web browser. Despite their widespread use in bioinformatics, biological web applications are usually implemented with no standard reutilisation guidelines in mind, hence BioJS was developed ^4^. BioJS code contains proper guidelines on how to use the components and how the API can be implemented to interact with other components.

BioJS is an open source JavaScript library of components for the visualisation of biological data on the web. Here we present *wigExplorer*, a standard, portable BioJS component designed to easily render wig data format files. *wigExplorer* can be integrated and controlled from other applications. To our knowledge, this is the first modular component to visualise wig data that complies with BioJS standards.

## The *wigExplorer* component

*wigExplorer* is fully integrated in the BioJS project. It follows the standards set by the BioJS registry ^5^, a centralised repository of BioJS components hosted at the European Bioinformatics Institute (EBI). Having *wigExplorer* in the BioJS registry is advantageous because it promotes i) easy discoverability for the component, ii) collaborative development with other members of the BioJS community and iii) reutilisation by third party applications. In the BioJS registry, component APIs are exposed, i.e., events and methods are defined and documented so that other BioJS components can interact with each other. By following these conventions, *wigExplorer* is able to interact with other components on the same web page, enriching the overall experience for the user. The code below shows how to incorporate *wigExplorer* into a web application. Only three configuration elements are needed: the target HTML element in which the component will be rendered, the background colour of the component, and the file path containing the wig data. Wig files contain minimalistic information of genomic data *wigExplorer* and can handle a large genomic region such as a chromosome (tested with a single file containing 12 chromosome with average length of 60 Mb), but this depends on the richness of the data rather than the length of the genomic region.



                    var instance = new Biojs.wigExplorer({
     target: "YourOwnDivId",
     selectionBackgroundColor: ’<
                    background
                    -colour>’,
     dataSet: "<path–to–file>"
});
                


*wigExplorer* uses D3.js, the data-driven documents JavaScript library ^6^, to generate graphical representations from wig data. D3.js handles the manipulation of the data documents, the reading of wig data as text format and their conversion to an area chart format. On the top right side, *wigExplorer* contains a dropdown to toogle between different references from the wig file. To control the visual aspect of the wig data, *wigExplorer* contains simple controls for zooming and panning. It is also possible to zoom and pan using provided API.



                    instance._updateDraw(start, end)
                


### Application

**Figure 1.**
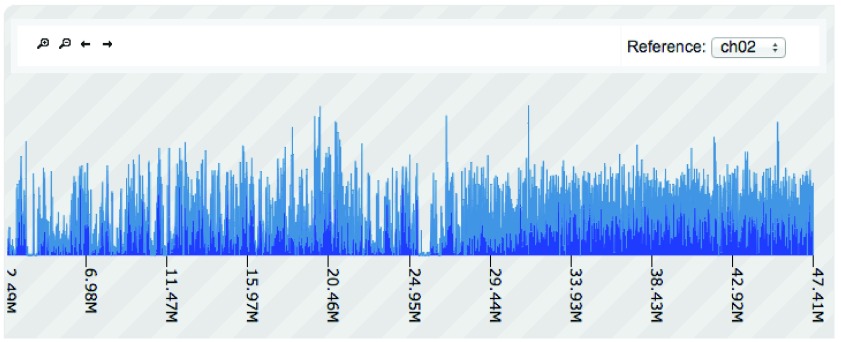
*wigExplorer* view of tomato variety Heinz chromosome 2. The top controls are designed to toogle between different references as well as zoom and pan. Peaks show SNP density of 1kb size bins. A change of SNP density can be observed around the 24M mark, with a slightly greater density of SNPs on the right, indicative of a potential introgression segment from another related species.

*wigExplorer* can be used to visualise genomic data in different ways. An application is shown in Figure 1, depicting single nucleotide polymorphism (SNP) density data from a genomic annotation in the tomato genome. Here chromosome 2 is zoomed in to show the genomic interval contained between position 2.5M and 47.5M. The SNP density data contained in the wig data file are presented as bins, where the Y axis indicates the number of SNPs contained in each bin. The screenshot shows a dramatic change in the density of SNPs just after the 24M bin mark of the chromosome, suggesting a potential boundary for an introgression segment introduced from a closely related tomato species. Other potential applications of *wigExplorer* may involve the visualisation of gene expression and alignment data.

**Figure 2.**
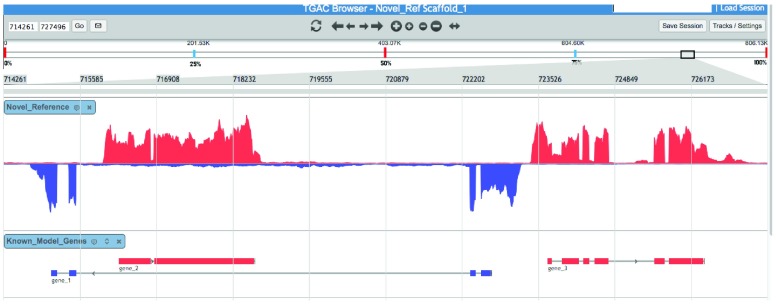
An example of *wigExplorer* integration using the TGAC Browser. The *wigExplorer* track shows read coverage in *Myzus* spp. for scaffold 1. Forward and backward strands are depicted in red and blue respectively. Evidence genes from a closely related species are displayed in the track below.

Third party browsers are also using *wigExplorer*. A screenshot of the TGAC Browser ^7^ is shown in Figure 2 using *wigExplorer* to depict *Myzus* spp. scaffold 1 zoomed in between regions 714K and 727K. Here strandspecific RNA-Seq paired-end read coverage is shown as a wig track. The track below shows a closely related annotated species gene set for comparison. This comparison suggests a potential gene extension in both forward and reverse orientation.

## Conclusions

The *wigExplorer* component provides a platform to visualise biological data in wig format. *wigExplorer* can be easily integrated with other web components or extended to provide new functionality. We expect this component to be particularly useful for visualisation in a variety of data types such as SNP density, alignments and gene expression. Like any other BioJS component, *wigExplorer* requires little technical knowledge for its utilisation.

## Software availability

Zenodo: wigExplorer, a BioJS component to visualise wig data_v2, doi: 10.5281/zenodo.8516 ^8^

GitHub: BioJS, http://github.com/biojs/biojs/releases/tag/v1.0;
