# Supplementary material for: wigExplorer, a BioJS component to visualise wig data
Source: F1000Res. 2016 Aug 9;3:53. Originally published 2014 Feb 13. [Version 3] doi: 10.12688/f1000research.3-53.v3 (PMC5054804; doi:10.12688/f1000research.3-53.v3)
Supplement: Peer review history

# Peer review: Referee response for version 3

**DOI:** 10.5256/f1000research.10145.r16472

**Published:** 2016-10-05

**Authors:** Chunlei Wu, Jiwen Xin

**Affiliations:** 1 Scripps Research Institute, La Jolla, CA, USA; 2 The Scripps Research Institute, La Jolla, CA, USA

This manuscript decribed a useful BioJS component for visualizing genomic tracks from a wig-formatted file. There are some major concerns raised by the reviewer:

- Demo code show “Unknown format detected” error. When clicking the demo code from the provided links to JSBin, Codepen and Plunker, all three have the same errors. This is also confirmed the same by running the demo code locally. But it can be fixed by changing the dataSet to:
  dataSet: "./wigExplorerDataSet5.txt"
  (was dataSet: "https://cdn.rawgit.com/anilthanki/biojs-vis-wigexplorer/master/snippets/data/wigExplorerDataSet5.txt")
  Even thought, there still are some javascript errors displayed on the browser console (using Google Chrome 54) as described below.
- The website http://biojs.io/d/biojs-vis-wigexplorer shows two javascript errors, even though the rendering of the widget looks fine. I confirmed that these errors are from the wigexplorer itself, as I can see the same errors in the console by running the demo code locally. This is the screenshot of the javascript errors:
http://imgur.com/a/sfWkK
- After several previous reviewers requested the actual code example for integrating WigExplorer into third-party applications like TGAC browser, you still did not provide much details of it. 
  The styling of the tracks in TGAC Browser screenshot does not look similar to the demo example, which does raise the concerns about how the integration was actually done in a real application, and how flexible a developer can control the look and feel of the application. A real code example should address those concerns.
- I also tried the demo code locally with two example wig files downloaded from NCI website: WashU (74MB) and Baylor (79MB). The WashU example file does load, but the track rendering is very slow, and the track control buttons (like zoom and move) are pretty much unusable. The Baylor example file does not load, also without any error message. These two files could be out of the file size range for reasonable rendering in this widget, a proper error msg should be displayed ideally.
- A minor suggestion, although the project website is provided under "Software availability" section, it would be helpful to provide the direct links to the demo code at the relevant text.

We have read this submission. We believe that we have an appropriate level of expertise to confirm that it is of an acceptable scientific standard, however we have significant reservations, as outlined above.

# Peer review: Referee response for version 2

**DOI:** 10.5256/f1000research.4634.r14186

**Published:** 2016-06-07

**Authors:** Stefan Thomas Lang

**Affiliations:** 1 Department of Molecular Hematology, Lund University, Lund, Sweden

I am no JavaScript developer myself and I have not tested the described functionality, but I already can think of applications in my own work if the package is able to not only show predefined chromosomes in a wig file, but subsets in a chromosome too.

 I will state all my issues like this first major one:

1. The link to your real example page (http://biojs.io/d/biojs-vis-wigexplorer) can only be found by reading your article, following to the rather unspecific GitHub link to the http://biojs.io web page. There one need to search for your wigexplorer - that is not acceptable! Make this link more prominent in the article and refer to the GitHub page as the main biojs.io GitHub page.
2. I can not approve this article, if your package can not display any user defined region in a wig file. After having checked the functionality on the hidden demo page I get the impression that this javascript package does ONLY visualize whole chromosomes from a wig file. The user can not change the visualized region by e.g. interactively zooming in or at least stating a displayed area in the javascript function call? But if your package can visualize a user defined area in a wig file this functionality needs to be highlighted in the article.

In addition I have several minor 'complains':

1. Can the package be used stand alone? If yes, please extend your code example to a minimal, working html page. It would be perfect to have the javascript library loaded from your own web server together with the example data. Much like on the hidden demo page, but as plain html plainly stated in the article.
2. You claim that your tool easily integrats into the third party TGAC Browser. Can you provide the example html there, too?

I hope I could add some additional points of view to your work and give a kind of positive feedback.

I have read this submission. I believe that I have an appropriate level of expertise to confirm that it is of an acceptable scientific standard, however I have significant reservations, as outlined above.

# Response

**Published:** 2016-08-03

**Authors:** Anil Thanki

**Affiliations:** The Genome Analysis Centre, UK

Dear Stefan

 We would like to thank you for reviewing the manuscript and making some positive comments that could be used for your application.

 Please find below our responses to your comments.

 1) The link to your real example page (http://biojs.io/d/biojs-vis-wigexplorer) can only be found by reading your article, following to the rather unspecific GitHub link to the http://biojs.io web page. There one need to search for your wigExplorer - that is not acceptable! Make this link more prominent in the article and refer to the GitHub page as the main biojs.io GitHub page.

 Answer: We can understand your concern, as this component was developed as a part of BioJS 1 and now BioJS is evolved to version 2, so some links are broken or redirected. We will update links in the article according. Link : http://www.biojs.io/d/biojs-vis-wigexplorer

 2) I cannot approve this article, if your package cannot display any user defined region in a wig file. After having checked the functionality on the hidden demo page I get the impression that this JavaScript package does ONLY visualize whole chromosomes from a wig file. The user cannot change the visualized region by e.g. interactively zooming in or at least stating a displayed area in the JavaScript function call? But if your package can visualize a user defined area in a wig file this functionality needs to be highlighted in the article.

 Answer: I can understand your issue here about visualising user defined region, wigExplorer was already had a capability of allowing user to move around and zoom in and out for visualising region of interest, but due to compatibility issue with BioJS2 buttons were not visible. Now we had fixed the issue and you will notice some controls on top left corner which gives user flexibility to visualise any region. It is also possible to visualise user defined area using API provided.

 3) Can the package be used stand alone? If yes, please extend your code example to a minimal, working html page. It would be perfect to have the javascript library loaded from your own web server together with the example data. Much like on the hidden demo page, but as plain html plainly stated in the article.

 Answer: Yes, this package has been designed to work standalone, I have set up some example on my personal GitHub page to prove that. Here is the link to stand alone example for wigExplorer: http://anilthanki.github.io/tools/biojs/wigExplorer/

 4) You claim that your tool easily integrates into the third party TGAC Browser. Can you provide the example html there, too?

 Answer: Yes, we believe that it's easy to integrate into any third party application and TGAC Browser uses wigExplorer to visualise users’ wig data. Here is an example explaining procedure to visualise your own wig data using TGAC Browser.

 Example:

1. Go to link http://browser.tgac.ac.uk/tgac-browser-demo/index.jsp?query=chr1&coord=4&&from=0&&to=5749886
2. Click on the upload icon on the right (before ‘Save Session’)
3. There is a link to example wig file, to save and upload to see example
4. Upload the same file to the ‘Drop files here’ region and close the upload box
5. You should see the uploaded wig file displayed with prefix of the filename you uploaded.

Regards,

 Anil Thanki

# Peer review: Referee response for version 2

**DOI:** 10.5256/f1000research.4634.r4977

**Published:** 2016-03-01

**Authors:** Phil Lord

**Affiliations:** 1 School of Computing Science, University of Newcastle, Newcastle, UK

The authors have provided answers to all my comments.

I have read this submission. I believe that I have an appropriate level of expertise to confirm that it is of an acceptable scientific standard.

# Peer review: Referee response for version 1

**DOI:** 10.5256/f1000research.3732.r3697

**Published:** 2014-03-07

**Authors:** Phil Lord

**Affiliations:** 1 School of Computing Science, University of Newcastle, Newcastle, UK

I have tried this piece of software and it does appear to do what it says and running the software was not too difficult. The link to the software on Zenodo in the Software Availability section ( Zenodo: wigExplorer, a BioJS component to visualise wig data, doi:10.5281/zenodo.77218) seems a bit pointless as the download bundle requires a large number of dependencies which are not included and for which paths have been hardcoded to

local machine locations. The authors should also include either a tag or a commit hash so that the version of the software in the download bundle can be identified in GitHub.

I ran the software from a GitHub checkout - here all the paths given in the documentation assume a specific configuration of directories (which is different from that of the GitHub repository). None of this is a show stopper, but it does make me rather doubt the validity of the statement: "Like any other BioJS component, *wigExplorer* requires little technical knowledge for its utilisation."

The article is, essentially, an advert with relatively little technical content. This is appropriate; there is little point including, for example, precise instructions for the use of wigExplorer as this article will be checkpointed and the code will become out of date. I would however have liked a bit more information on the expected performance of the software: for instance, how big a chromosome can it visualize? How manipulable is the visualisation?

The writing in the article is okay, but there are a few parts which could do with improvement. For example, in the first paragraph of the Introduction section, the statement "biological web applications are usually implemented with no standard reutilisation guidelines in mind, hence BioJS was developed" I am not at all sure what "reutilisation guidelines" means with respect to code base. Do the authors just mean a defined and documented API? In which case, they should probably say this.

There are also a couple of places which could do with simplification. In the second paragraph of the Introduction section:"To our knowledge, this is the first modular component to visualise wig data

that complies with BioJS standards." This statement could be seen as a caveat, and I think the authors could risk saying "This is the only BioJS component to show wig data". Also in the second paragraph of the Application section the statement "We are aware that third party browsers are also using *wigExplorer*." could perhaps be simplified to "Third party browsers are also using *wigExplorer*".

I have read this submission. I believe that I have an appropriate level of expertise to confirm that it is of an acceptable scientific standard, however I have significant reservations, as outlined above.

# Response

**Published:** 2014-05-23

**Authors:** Anil Thanki

**Affiliations:** The Genome Analysis Centre, UK

We would like to thank Phil for taking the time to review the manuscript. 

Please find below our responses to your comments

1) The link to the software on Zenodo in the Software Availability section ( Zenodo: wigExplorer, a BioJS component to visualise wig data, doi:10.5281/zenodo.77218) seems a bit pointless as the download bundle requires a large number of dependencies which are not included and for which paths have been hardcoded to local machine locations. The authors should also include either a tag or a commit hash so that the version of the software in the download bundle can be identified in GitHub.

Answer: We have uploaded a new version of the code to Zenodo with dependencies provided ( http://dx.doi.org/10.5281/zenodo.8516). This new version is also included in BioJS Release 1.0 https://github.com/biojs/biojs/releases/tag/v1.0

2) It does make me rather doubt the validity of the statement: "Like any other BioJS component, *wigExplorer *requires little technical knowledge for its utilisation."

Answer: We understand that there are a number of required dependencies for this component, and not including these in the component’s distribution made it harder to install and utilise. As above, we have included these dependencies as well as sample datasets, so simply unzipping the distribution is sufficient to install and start using the component.

3) The article is, essentially, an advert with relatively little technical content. This is appropriate; there is little point including, for example, precise instructions for the use of wigExplorer as this article will be checkpointed and the code will become out of date. I would however have liked a bit more information on the expected performance of the software: for instance, how big a chromosome can it visualize? How manipulable is the visualisation?

Answer: *F1000Research* requires a working copy of the code when article get published (for archival purposes) so, we have uploaded current version of code to zenodo ( https://zenodo.org/record/8516#.U334LlhdV0s) so this copy of code will be same though main code can be updated. So this article will always point to the version of the code that it describes, which is stored in zenodo. And on the performance issue, in this case size of the file matters rather than size of genomic region. Because size of data depends on depth of the genomic information, though we tried to make an effort to make it clear in manuscript. In first paragraph of wigExplorer component section, “Wig files contains minimalistic information of genomic data, *wigExplorer *can handle large genomic region like a chromosome (tested with a single file containing 12 chromosome with average length of 60 Mb), but it depends on depth of data rather than length of genomic region.”

4) The writing in the article is okay, but there are a few parts, which could do with improvement. For example, in the first paragraph of the Introduction section, the statement "biological web applications are usually implemented with no standard reutilisation guidelines in mind, hence BioJS was developed" I am not at all sure what "reutilisation guidelines" means with respect to code base. Do the authors just mean a defined and documented API? In which case, they should probably say this.

Answer: Here by using the word "reutilisation guidelines”, we mean that BioJS components can be easily implemented and can interact with each other with standard API. We have added a statement explaining this in the last line of the first paragraph of introduction that “BioJS code contains standard guidelines that how to use components and how API can be implemented to interact with other components.”

5) There are also a couple of places, which could do with simplification. In the second paragraph of the Introduction section:" To our knowledge, this is the first modular component to visualise wig data that complies with BioJS standards." This statement could be seen as a caveat, and I think the authors could risk saying "This is the only BioJS component to show wig data". Also in the second paragraph of the Application section the statement "We are aware that third party browsers are also using *wigExplorer*." could perhaps be simplified to "Third party browsers are also using *wigExplorer*". I have read this submission. I believe that I have an appropriate level of expertise to confirm that it is of an acceptable scientific standard, however I have significant reservations, as outlined above.

Answer: We have reformatted the first statement to “Currently, this is the only BioJS component to show wig data” and we have changed the second statement as reviewer suggested, “Third party browsers are also using *wigExplorer”*

# Peer review: Referee response for version 1

**DOI:** 10.5256/f1000research.3732.r3699

**Published:** 2014-02-25

**Authors:** Robert Buels

**Affiliations:** 1 JBrowse, University of California, Berkeley, CA, USA

The manuscript is well written, but it does not accurately describe the software in question.

I installed the software on my machine, and evaluated its behavior under several scenarios that I think would be commonly encountered by people using it. The major issues I found with the software are:

1. *wigExplorer* does not provide any useful error messages when it fails to fetch, or fails to parse, the file specified in its `dataSet` option.  This can be a major issue for prospective users (i.e. installers) of the software.
2. Also, when I installed it and ran it via the included TestwigExplorer2.html, the "top controls ... designed to scroll sideways" described in the manuscript were not shown. To rule out any problems with the way I installed it (caused by missing stylesheets or the like), I inspected the browser's (Google Chrome 32) document object model (DOM) using its built-in development tools, and verified that the controls were not even being placed in the DOM by the code itself.  I didn't debug any further.
3. Most importantly, and I think this is a show stopper: wigExplorer does not actually support the Wiggle format as set forth by UCSC at http://genome.ucsc.edu/goldenPath/help/wiggle.html.  It supports a limited tab-separated value format, which bears some resemblance to BedGraph format, but is not BedGraph either.  So: it is named for a standard data format that it does not support, and the data format that it actually supports is not a standard format.

Unfortunately, I cannot approve this manuscript as it is currently written. It does not accurately describe the wigExplorer software.

I have read this submission. I believe that I have an appropriate level of expertise to state that I do not consider it to be of an acceptable scientific standard, for reasons outlined above.

# Response

**Published:** 2014-05-28

**Authors:** Anil Thanki

**Affiliations:** The Genome Analysis Centre, UK

Dear Robert

We would like to thank you for taking the time to review the manuscript.

Please find below our responses to your comments.

*1) wigExplorer* does not provide any useful error messages when it fails to fetch, or fails to parse, the file specified in its `dataSet` option.  This can be a major issue for prospective users (i.e. installers) of the software.

Answer:  After considering this comment we have added more informative error messages, depending on the errors that can occur while running the component, e.g. “Unknown format detected”,  “No data for selected reference”, “File not found”, etc.  This will help make any installation problems clearer to users during installation.

2) Also, when I installed it and ran it via the included TestwigExplorer2.html, the "top controls ... designed to scroll sideways" described in the manuscript were not shown. To rule out any problems with the way I installed it (caused by missing stylesheets or the like), I inspected the browser's (Google Chrome 32) document object model (DOM) using its built-in development tools, and verified that the controls were not even being placed in the DOM by the code itself.  I didn't debug any further.

Answer: Outside of the BioJS registry environment, the component’s CSS behaviour changes, which resulted in disappearing controls. We have reformatted the CSS for this component and this is now fixed.

3) Most importantly, and I think this is a showstopper: wigExplorer does not actually support the Wiggle format as set forth by UCSC at http://genome.ucsc.edu/goldenPath/help/wiggle.html.  It supports a limited tab-separated value format, which bears some resemblance to BedGraph format, but is not BedGraph either.  So: it is named for a standard data format that it does not support, and the data format that it actually supports is not a standard format.

Answer: We thanks Robert t raise this concern and we have rewritten the code and now the component fully supports the standard fixedStep and variableStep wig file format (as set out by UCSC) with span information and multiple references in one file if available.
